# CT-based fractional flow reserve: development and expanded application

**DOI:** 10.21542/gcsp.2021.20

**Published:** 2021-10-30

**Authors:** Ryo Torii, Magdi H. Yacoub

**Affiliations:** 1 Department of Mechanical Engineering, University College London, London, UK; 2 Department of Surgery and Department of Cardiology, Aswan Heart Centre, Magdi Yacoub Heart Foundation, Aswan, Egypt; 3 Magdi Yacoub Institute, Harefield Heart Science Centre, Harefield, UK; 4 National Heart and Lung Institute, Imperial College London, UK

## Abstract

Computations of fractional flow reserve, based on CT coronary angiography and computational fluid dynamics (CT-based FFR) to assess the severity of coronary artery stenosis, was introduced around a decade ago and is now one of the most successful applications of computational fluid dynamic modelling in clinical practice. Although the mathematical modelling framework behind this approach and the clinical operational model vary, its clinical efficacy has been demonstrated well in general. In this review, technical elements behind CT-based FFR computation are summarised with some key assumptions and challenges. Examples of these challenges include the complexity of the model (such as blood viscosity and vessel wall compliance modelling), whose impact has been debated in the research. Efforts made to address the practical challenge of processing time are also reviewed. Then, further application areas—myocardial bridge, renal stenosis and lower limb stenosis—are discussed along with specific challenges expected in these areas.

## 1. Background

Computationally-derived fractional flow reserve (FFR) is one of the most promising non-invasive tools ready for expanded use in clinical cardiology. The use of FFR during coronary angiography has been successful in improving patient selection for revascularization^1^. However, this method is invasive and cannot be repeated readily with follow-up of patients with coronary disease. This highlights the need for developing reliable non-invasive methods for measuring FFR. We here review the development and potential applications of CT-based measurements of FFR (CT-FFR).

## 2. Technology and efficacy

The key technology behind CT-FFR is patient-specific modelling of blood flow^2,3^. This essentially requires three key elements: (1) anatomical model of the vasculature, (2) mathematical equations to describe blood flow (including required parameters such as blood viscosity), and (3) boundary conditions, *e.g.*, reference flow and pressure conditions. Each of these is described below.

### 2.1 Anatomical model of the vasculature

**Figure 1.**
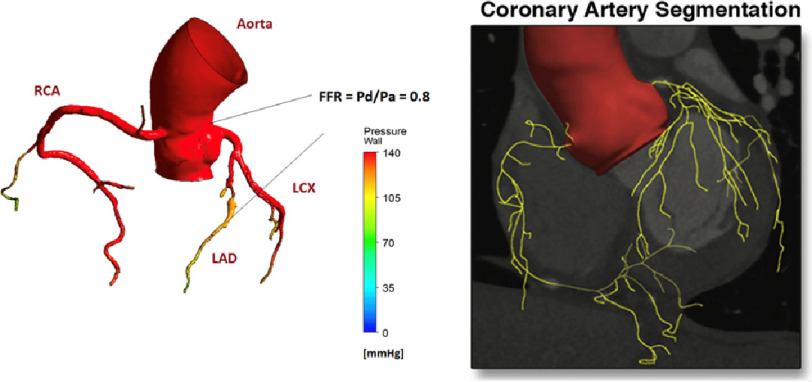
Typical coronary tree structure reconstructed from CT images. Left: direct reconstruction from raw CT images^13^, right: the centreline of an arterial tree reconstructed using a machine-learning enhancement^5^.

In the process of CT-FFR analysis, the geometry of a coronary vascular tree is reconstructed in 3D, from CT coronary angiograms (CTCA). An example of a reconstruction is shown in Figure 1, showing the aortic root, left and right coronary vascular trees, including a stenosis.

A common image segmentation process involves thresholding of the CT image intensity to allow for the lumen border to be delineated, and a 3D vascular (lumen) model to be obtained. This process is limited by the spatial resolution of CTCA, which is typically around 0.5 mm (both in plane, *i.e.*, pixel size, and slice interval). Considering a standard coronary artery diameter of 3–4 mm, typical CTCA resolves the vessel diameter in 6–8 pixels and most of the CT-based coronary tree models therefore only include the 1st generation of coronary artery branches from the 3 main coronary vessels.

Because drastic improvement of CTCA resolution is not expected, at least in the immediate future^4^, efforts have been devoted to improving image processing algorithms, such as the use of resolution enhancement and machine learning.

Min et al. demonstrated finer details of coronary tree structure segmented using their sub-voxel enhancement approach (Figure 1^5^). Whilst the application of machine-learning techniques to cardiac CT has been explored^6^, quantitative assessment of its impact, especially on CT-FFR, has not been clearly described. However, overall, the importance of anatomical model in patient-specific CFD is well documented from the earliest era of blood flow simulations^7^ and also in terms of its impact on FFR computation^8^.

Anatomical models of coronary vessels can be reconstructed from images acquired with other modalities. Cardiac magnetic resonance (CMR) imaging is an ideal modality as it requires neither radiation, nor nephrotoxic contrast agent, but the spatial resolution (∼1 mm/voxel) is less than CTCA^9^.

On the other hand, X-ray angiograms have been utilised in computational prediction of FFR, *e.g.*, in vFFR^10^ and QFR^11^ approaches. Whilst this approach has been shown to be effective in reliably predicting FFR, it requires the use of an invasive procedure. Information on this approach has been reviewed elsewhere^12^.

### 2.2 Mathematical equations to describe blood flow

Typically, equations of fluid motion—the incompressible Navier–Stokes equations—are solved on computers as the basis of computational fluid dynamics (CFD) analysis. Here, we describe only some key assumptions involved, while further information on the model details can be found in the literature^2,3^.

The coronary arterial network is a three-dimensional (3D) structure and many CT-based FFR computations, including HeartFlow FFR_CT_, are conducted in 3D, which is based on solving the full 3D version of the above-mentioned mathematical equations. This normally requires hours of computational time, in addition to the pre- and post-processing of the data (computational time is discussed in more details later in Section 3). At the same time, attempts have been made to approximate the computation using 1-dimensional model of blood flow, in which a 1D version of the Navier–Stokes equations are solved. This approach—often called a reduced-order model—is less time-consuming and is adopted in Siemens cFFR^14^ and Toshiba CT-FFR^15,16^ platforms. Comparisons of 3D and 1D FFR computation have demonstrated similarity of the results between those two approaches^17^, and also for a complex aortic flow^18^. Thus a reduced order model may be chosen, depending on the required model output.

It is widely accepted that the density and viscosity of blood can be assumed to be constant across space and time. The density of blood is shown to be homogeneous across individuals, 1050 kg/m^3^ (95% CI [1048–1054] kg/m^3^)^19^. The viscosity of blood is known to be shear-thinning which may affect the blood flow pattern and eventually FFR. A number of non-Newtonian blood viscosity models, such as the Quemada model, allow for the implementation of shear-thinning characteristics and adjustment of the viscosity *vs.* shear rate relationship, also based on patient’s haematocrit^20^.

Inclusion of other blood properties, such as blood protein, are less common and only a limited number of models are readily available (*e.g.*, Walburn-Schneck model^21^). At the same time, a simple estimation shows that the mean shear rate in coronary arteries (1 ml/s of blood flowing through a 3 mm diameter vessel) is approximately 189 s^−1^. Experimental data on human blood viscosity shows a plateau of viscosity in shear rate >100 s^−1^, which indicates that the assumption of constant viscosity is acceptable^22^, especially around a stenosis, where shear rate in the blood flow is expected to be even higher.

Another typical assumption is that the vessel wall is approximated as rigid. By this, the model ignores two potentially contributing factors: wall compliance and cardiac-induced wall motion. The former was examined by comparing computational results from 3D simulations assuming rigid vessel wall, and from 1D simulations incorporating wall compliance^17^. Their results demonstrated a difference of 0.00  ± 0.03 between FFRs based on 3D rigid wall and 1D compliant wall models. Although this should account for the difference between 3D and 1D simulations, it suggests that the impact of wall compliance is not significant. Additionally, FFR is commonly taken in diastole when FFR becomes the maximum FFR within a cardiac cycle^23^. Here, the wall deformation is expected to be smaller due to lower diastolic pressure hence the impact of wall compliance on FFR is likely to be even smaller.

The impact of cardiac-induced motion is an open question, with examinations focusing more on wall shear stress^24^, rather than pressure or FFR. The dynamic variation of vessel wall centreline curvature could alter the FFR dynamically, and this is an interesting topic to be explored, especially in some specific pathologies such as myocardial bridge, which is discussed later in this review.

### 2.3 Boundary conditions

To reliably predict the blood pressure in a patient’s coronary arterial tree, it is important to set the flow and pressure conditions to represent the patient. It has been shown that if the boundary conditions are well defined, ideally with intravascular measurement of pressure and flow velocity, the computationally predicted FFR in a short segment of blood vessel closely agrees with the invasively measured FFR^25^. However, in CT-based FFR computations, such data is not likely to exist because of the non-invasive nature of the process, and boundary conditions need to be estimated, making the best use of limited information about patient’s flow and pressure.

Taylor et al.^3^ employed lumped parameter (electric circuit) model of the heart and the peripheral vasculature to address the challenge of boundary conditions, both inflow from the heart and outflow to downstream coronary microvasculature. They adjust the lumped parameter model such that the mean aortic pressure matches the patient’s own brachial pressure, and the aortic and coronary outflow match those estimated from allometric scaling law. A similar adaptive modelling approach was taken by Kruk et al., by coupling a simpler model of coronary and systemic circulation that is tuned based on patient-specific blood flow and pressure from clinical recording^14,26^.

Ko et al. took a different approach to estimate blood pressure based on deformation of blood vessel, estimated from 4D CT image sets, and vessel stiffness, which subsequently allows estimation of blood flow in the aorta and coronary tree^15^. At the same time, when 4D CT image sets are available, aortic inflow can be estimated by taking the difference of the left ventricular cavity volumes between end systolic and end diastolic phases, as the stroke volume^13^.

In relation to the outflow condition of the coronary tree, one of the debated topics is the model of hyperaemic condition. All of the above-mentioned works assume constantly increased flow under hyperaemia, either in terms of peripheral resistance reduction to 24–30% of the rest, or flow increase of 4.0–4.5 fold^27^.

It has also been assumed that the response to adenosine is the same across patients and across different coronary territories. However, it has been reported that the drug (dipyridamole)-induced vasodilatory response is not homogeneous even among normal subjects without cardiac disease^28^. Our study on the impact of outflow conditions indicated that it could considerably affect the predicted FFR more in anatomically severe stenosis, by altering the flow distribution in different coronary vessels, and possibly alter the FFR across the diagnostic threshold^9^. Outflow boundary condition could therefore be one of the areas that could improve CT-based FFR prediction, potentially with new sophisticated models of myocardial blood perfusion^29^.

### 2.4 Clinical efficacy of the method

**Figure 2.**
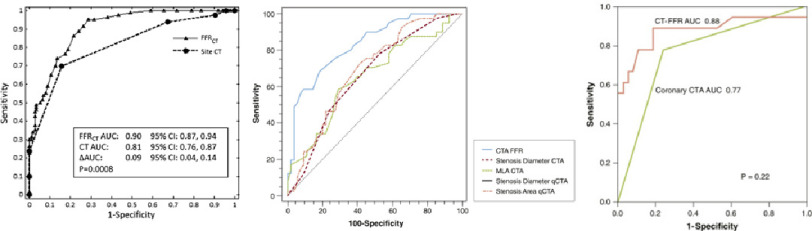
ROC curves of various CT-based FFR computation methods. Noorgaard et al.^30^ (HeartFlow FFR_CT_, left), Kruk et al.^14^ (Siemens cFFR noted as CTA FFR, middle) and Ko et al.^15^ (Toshiba CT-FFR, right). The AUC of CTA FFR in the mid panel is 0.835.

The efficacy of CT-based FFR computation is well documented and some representative ROC curve analysis results are shown in Figure 2. Since our aim is to focus more on the technical aspect, we only briefly describe a typical result from a number of clinical trials. In Norgaard et al., the per-patient sensitivity and specificity of HeartFlow FFR_CT_ to identify myocardial ischemia, were reported to be 86% and 79%, respectively, which were superior to coronary CT angiogram (CTCA) alone (sensitivity and specificity 94% and 34%, respectively)^30^. This is also reflected in the difference in area under the curve (AUC) which was 0.90 for FFR_CT_ and 0.81 for CTCA, based on invasive FFR as the gold standard. Along with the number of other evidences demonstrating its efficacy, and due to its non-invasive nature, HeartFlow FFR_CT_ gained its current position in clinical practice, for example in the UK, based on a recommendation in UK NICE guidelines which compared HeartFlow FFR_CT_ to other CCTA and MRI based non-invasive approaches^31^.

The efficacy of other CT-based FFR computation approaches has been demonstrated more in a research context. Diagnostic accuracy of Siemence cFFR methodology per patient was documented as 75.6% sensitivity, 71.4% specificity and AUC 0.835 in a cohort of 90 patients^14^.

The accuracy of Toshiba CT-FFR was examined in a cohort of 30 patients and shown to have 77.8% sensitivity, 86.8% specificity and AUC 0.88 after calibration of FFR cut-off value to improve the sensitivity and specificity^15^. These diagnostic accuracies are also shown to be superior to other non-invasive measures, especially CTCA alone (Figure 2). Though these technologies would require further evaluation in a larger group of patients, in principle, CT-based FFR computation has been shown to have high diagnostic values in different forms of implementation.

### 2.5 Additional advantage of computational approach

Computation of FFR produces a rich set of information. The direct output of the CFD process is blood pressure and flow velocity at any point in the region of interest. FFR is derived from the blood pressure, and the obvious advantage of computationally-derived FFR is the ease of repeated and/or pullback “measurements” which can be particularly effective in assessing serial legions^32^.

Derivation of other haemodynamic parameters, such as wall shear stress (WSS)—sometimes called endothelial shear stress—is another advantage. WSS and its derivations have been explored as a marker to predict progression of atherosclerotic disease^33^, and WSS-based parameters have been shown to be effective markers to predict disease progression in patients with borderline negative FFR^34^. There are also some new parameters proposed such as axial plaque stress^35^ and a combination of various computationally-derived parameters could reinforce FFR-only assessment of the lesion, by taking the full advantage of the rich computational data output.

Finally, it should be mentioned that computational simulation can be used to predict post-PCI flow characteristics and FFR, as demonstrated by Modi et al.^36^. In their study of 24 vessels with serial stenoses, post-PCI prediction of FFR, using their new and simplified mathematical model for a shorter turnaround time, was shown to match invasively measured post-PCI FFR better (7% error with FFR difference of 0.01 ± 0.05). This proof-of-concept study was followed up in their Benefits of Obtaining information or planning With noninvasive FFR _CT_ prior to Invasive Evaluation (BOWIE) study and in their latest presentation, they reported that the tool suggested a change of diagnostic plan in 45% of their patient cohort^37^. As what-if analysis is one of the strengths of computational modelling, a wider use of CT-based FFR computation in this fashion is expected in the future, with more rigorous validations.

## 3. Challenges and new application areas

### 3.1 Analysis platform and speed

In the previous section, we described that CT-based FFR computation has successfully been applied in various clinical scenarios, with some technical challenges associated with assumptions in the model. More practical questions relate to where and who does the analysis, and the speed of computation.

The HeartFlow FFR_CT_ analysis is conducted at a US-based data processing centre and the turnaround time of the service is noted as 24 h in a clinical trial description from 2012^38^ and 48 h in the NICE guideline^31^. The time includes data transfer, data processing and quality assurance, of which the actual processing time is reported as 1–4 h per case^30^. Whilst this approach offers a rigorous quality control, which is typically time consuming, even with trained data processing technicians, other groups take a different approach. Both Kruk et al.^14^ and Ko et al.^15^ (works associated with Siemens cFFR and Toshiba CT-FFR, respectively) opted for computations to be done on a local desktop computer. Localisation of analysis is quicker and may be more clinically viable in terms of assessment time. At the same time, quality of the analysis output may depend on the skill of the local operator and could introduce uncertainty and/or variability in the analysis.

The reduced order (1D) approaches adopted in Siemens cFFR and Toshiba CT-FFR, introduced earlier in Section 2, produces computational results more quickly. The former is reported to complete the whole process in 23.9 ± 11.2 min^39^ and the process of the latter is completed in 27.07 ± 7.54 min^15^, both on standard desktop workstation without significantly sacrificing accuracy, as shown in Figure 2. However, it should be noted that the Siemens and Toshiba methods has only been validated in relatively small cohorts (*n* = 90 and 30, respectively). Another study directly comparing 3D simulation and 1D reduced-order simulation of flow in the coronary arteries of 20 patients’ reported mean execution time of 27.2 h (3D) *vs* 0.09 h (1D), with extremely good agreement in the FFR between the two methods (difference between FFR_3D_ and FFR_1D_ = 0.00 ± 0.03)^17^.

To accelerate the process further, the use of machine learning (ML) has been explored, as seen in many other applications in biomedicine. Siemens cFFR was developed further in this direction, and its ML version appears the most in the literature^40,41^ as well as in a clinical trial database^42^. The ML-predicted FFR was produced in approximately 80 times shorter computational time (196.3 ± 78.5 s for CFD and 2.4 ± 0.44 s for the ML, both on a standard desktop PC) with FFR values and diagnostic accuracies similar to the reduced-order (1D) CFD-based FFR^40^. Its efficacy has also been shown in terms of its capability to stratify patients with MACE in a 2-year window^41^.

The drastic acceleration of the computational time by ML is deemed more beneficial if applied to more time-consuming 3D simulations, whose output can be more informative as described earlier. However, the number of attempts made on predicting 3D-simulation-based FFR using ML is limited, although prediction of 3D WSS can be found^43^. The time-consuming nature to generate sufficient training dataset with 3D simulation is a potential reason behind that, although this could be a worthy investment considering the benefit of obtaining FFR (and relevant) data equivalent to 3D simulation output in a very short time.

A more extreme form of ML-based prediction of FFR is to train a ML model directly using pairs of raw CT data and invasive FFR. Kumamaru et al. attempted this using pairs of those data for 131 patients, of which 20 were in the validation data set, and the diagnostic accuracies were reported to be 69.2% sensitivity, 66.2% specificity and AUC 0.78, using invasive FFR as the reference^44^. The diagnostic indices are lower than for the previously mentioned approaches, which could be improved with an improved intermediate step (currently, they use the morphology of stenosis segmented from CTCA).

Considering the demand of analysis speed in clinical environments, the use of reduced order model and/or ML may become more mainstream than 3D computation. At the same time, careful consideration is required in selecting the acceleration strategy, depending on the available input data and its quality (control), and the required output information.

### 3.2 Potential new application areas

Stenosis can be found in many different parts of the cardiovascular system. And blood flow computations using 3D patient-specific vascular models have been applied to a vast variety of anatomical locations in human cardiovascular systems. However, the application of CT-based FFR calculation is still limited to the assessment of coronary atherosclerotic narrowing. This is likely because the evidence for the efficacy of FFR, or any haemodynamic parameters, in assessing other pathologies is not as strong as the efficacy of FFR in guiding treatment for coronary stenosis, which has been demonstrated by many, including the landmark DEFER and FAME trials^1,45^. The beauty of CT-based FFR computation is the fact that it simply replaces a well-established clinical indicator with a non-invasive alternative.

We discuss potential future applications of CT-based FFR based on the recent exploration of invasive FFR application, along with expected technical challenges specific to the computational approach.

### 3.2.1 Myocardial bridge (MB)

The application of FFR to assess MB was first attempted by Escaned et al. in 2003^46^. With the stress induced by dobutamine, they assessed haemodynamic relevance of MB in 12 patients, using diastolic FFR, with FFR = 0.76 as the cut-off value that was proposed as the cut-off for static stenoses in diastole^23^.

**Figure 3.**
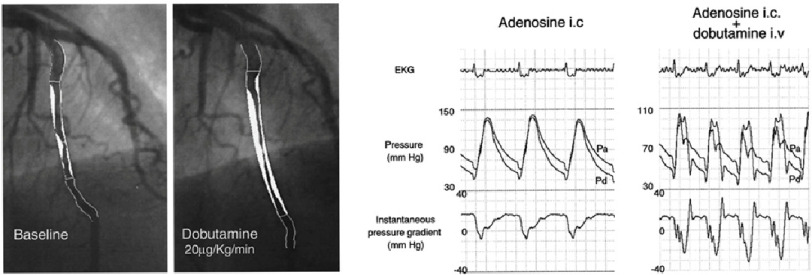
X-ray angiogram showing coronary artery morphology change under dobutamine stress (left) and intravascular pressure measurement across MB section under dobutamine and adenosine stress (right), reported by Escaned et al.^46^. Pa, aortic pressure; Pd, intracoronary pressure distal to the MB.

Escaned et al. reported that diastolic FFR is significantly lower than cycle averaged FFR, and that diastolic FFR under dobutamine stress is the best indicator for the haemodynamic significance of MB. They demonstrated, using intravascular pressure measurement, that the systolic pressure difference across the MB segment is reversed, *i.e.,* distal blood pressure is higher than the proximal blood pressure (Figure 3). This is due to dobutamine-enhanced systolic compression of myocardium. Subsequently, a larger pressure difference across the MB segment was observed in diastole compared to the rest, which lead to the conclusion that diastolic FFR under dobutamine stress is the most severe measure, hence the most adequate way, to assess the haemodynamic severity of MB. The effect of dobutamine infusion on FFR in MB was later confirmed by Hakeem et al.,^47^ though with a higher dose than Escaned et al., and in a smaller number of patients.

In principle, this assessment can be replicated using CT-based FFR approach. An obvious challenge is to capture the dynamically changing vessel morphology with CT. Since the typical number of time instances in 4D Cardiac CT is 10 and the instances are at equal time interval (which could go up to 20-time instances^48^), characteristic time points of myocardial contraction may be missed. However, if diastolic FFR is focused as suggested by Escaned et al., this issue may not impact the FFR estimation significantly. Diastolic measurement would also alleviate the concern of motion artifact in cardiac 4D CT.

The real challenge in modelling the flow in MB patients would be to reflect the diastolic flow increase, likely to be due to the systolic compression, in addition to the effect of adenosine. This is the key to the elevated pressure difference across the MB and plays a significant role, although such information would not be available from non-invasive measurement and an additional model, mathematical and/or empirical (statistical), would be required.

More recently, the use of instantaneous wave-free ratio (iFR) was suggested for assessing severity of MB by Tarantini et al.^49^. This could also be derived computationally, using the same computational approach with CT-based FFR computation but without considering the effect of adenosine. However, Tarantini et al. indicated that further studies are still needed to confirm the clinical value of iFR, which is the same challenge with FFR.

Assessment of MB severity using CT-based FFR has not been described yet, although the technology has been used to predict atherosclerotic plaque formation in MB patients using ML-accelerated version of Siemens cFFR technique^50^. It appears that MB is an uncharted territory to apply CT-based FFR computations.

### 3.2.2 Renal artery stenosis

Variants of FFR have been used to assess the severity of renal artery stenosis as summarised in the review by van Brussel et al.^51^. Their systematic review included 6 studies examining the efficacy of hyperaemic haemodynamic indices in guiding the therapeutic strategy on renal artery stenosis (the number of patients ranged from 17–62).

**Figure 4.**
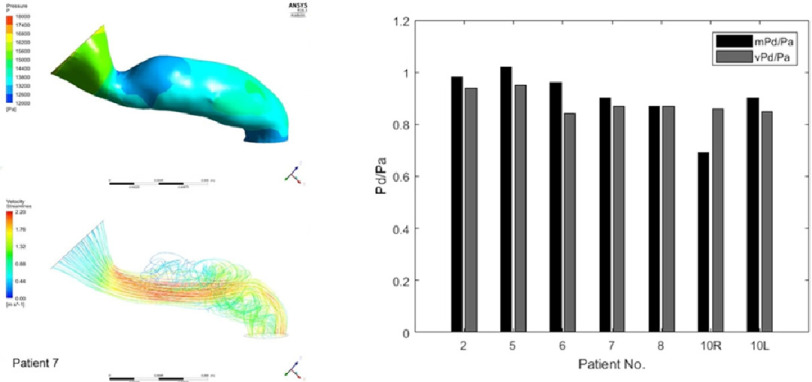
Example result of computationally predicted blood flow and Pd/Pa in renal artery stenoses^54^. Three-dimensional model and flow pattern example (left) and a comparison between measured and computed Pd/Pa (right).

**Figure 5.**
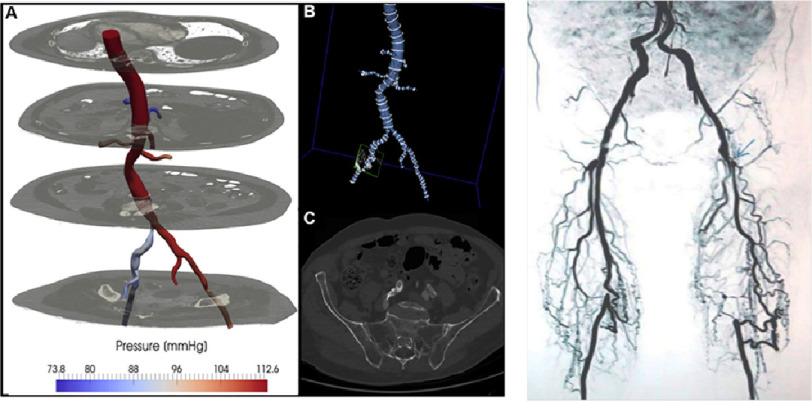
CT-based FFR computation of aortoiliac stenosis (left)^59^ and collateral vessel network around common femoral artery stenoses (right)^62^.

The review has shown that the use of renal FFR (rFFR) is promising in terms of differentiating responders from non-responders of percutaneous transmural renal angioplasty with stent placement (PTRAS). However, the actual threshold rFFR value is yet to be established. Additionally, integration of the microvascular response (renal flow reserve: RFR) with rFFR was discussed, similar to the combination of FFR and coronary flow reserve (CFR). However, that is still conceptual and only limited data is available on difference of hyperaemic response in renal microvasculature due to co-morbities (*e.g.*, diabetes^52^).

Since the renal arteries are relatively large (diameter ∼5 mm) in comparison to the resolution of CT (∼0.5 mm/pixel) and are static, they are expected to be well depicted in CT images. This allows reconstruction of 3D arterial model as shown in Figure 4 (left panel) and makes this application relatively straightforward in terms of applicability of CFD.

The application of computational FFR, sometimes referred as Pd/Pa in this context, was attempted almost 2 decades ago based on CFD and contrast-enhanced MR images^53^, which is even less invasive compared to the CT-based techniques. More recent attempts using CT images have successfully demonstrated a good quantitative agreement of Pd/Pa values between computational prediction and invasive measurement (Figure 4)^54^.

One challenge expected here is the definition of boundary conditions. In the MR-based study, phase-contrast MR was utilised to obtain inflow velocity from the aorta to the renal arteries^53^, which is a common sequence and straightforward to use. CT-based analysis requires an additional measurement or model to obtain inflow condition. A 1-dimensional model of systemic circulation was used by Mandaltsi et al.^54^.

Additionally, although a model of outflow condition would be needed irrespective of the imaging modality, that would be more straightforward compared to the coronary system since the stenosis is expected primarily in the main renal artery that feeds the entire kidney, *i.e.,* heterogeneity of ischaemic region is less likely in renal vasculature than coronaries. In the case of a renal artery stenosis, a simple resistance model could be used.

Furthermore, the computational approach has been utilised in the context of predicting the efficacy of stenosis removal^55^. All the previous works mentioned above, both on clinical efficacy and computational prediction of Pd/Pa, indicate that studies are expected, in the near future, on application of CT-based FFR calculation to renal artery stenosis, including demonstration of its clinical significance.

### 3.2.3 Lower-limb ischaemia (peripheral artery disease)

Assessment of lower limb (aortoiliac) stenosis severity using FFR (-equivalent parameter) is a newer application area than MB and renal artery stenosis. The first attempt was made by Banerjee, et al. investigating an association between invasively-measured pressure difference (gradient) across a lesion and walking impairment^56^. They found that using a threshold value of 11 mmHg, the pressure difference can predict walking impairment with 71% sensitivity and 100% specificity.

The efficacy of FFR in assessing below the knee stenotic and ischaemic lesions are also explored by Ruzsa et al.^57^, in which a significant correlation between FFR and transcutaneous O_2_ pressure, measured by a laser Doppler system, was demonstrated. A more detailed summary can be found in a review by Mangi et al.^58^, but it appears that the use of FFR in assessing lower limb stenosis is still being established. An additional question in lower limb ischaemia may be the ease of access to the diseased site compared to the prior examples, *i.e.,* coronary and renal arteries, and the efficacy and practicality of computationally-derived indicators will be scrutinised against direct (and non-invasive) measurements such as ultrasound.

Similar to renal artery examples, application of CT-based FFR computation to lower-limb arteries is expected to be relatively straightforward because the diameter of major lower-limb arteries is large enough (>5 mm) in comparison to typical spatial resolution of CT (∼0.5 mm/pixel) and no major vessel motions (*e.g.*, cardiac- or respiratory-induced) is expected.

Ward et al. computed CT-based FFR in 7 patients with aortoiliac obstructive disease (Figure 5 left) and showed that the agreement of FFR between the computational predictions and *in vivo* measurement was excellent. The average difference of FFR is 0.136 and the area under the curve is 1.0, taking *in vivo* measurement based diagnostic as the gold standard^59^. The high level of agreement was achieved even though they applied uniform flow of 8.0 L/min, including the hyperaemic flow increase, across the patients and computations under steady flow assumption.

These promising results suggests that the application of CT-based FFR could be explored more, but there is no large-scale study or clinical trial on the horizon. The CFD-based studies so far concentrate more on predicting restenosis (mechanobiological adaptation) by quantifying haemodynamic shear stress acting on the endothelium^60^, including the effect of leg flexion^61^.

One of the potential reasons behind that is the development of collateral vessel network that is often observed in aortoiliac/femoral arterial obstruction^62^ (Figure 5 right). The presence of collateral vessel alleviates the high pressure drop across a stenosed lesion, but to capture the small collateral arteries in CT images is a challenge. However, overall, lower limb ischaemic disease is a promising application of CT-based FFR technology because this routine clinical diagnosis involves many (non-invasive) haemodynamic measurements that can be used for boundary conditions of CFD modelling^63^, among which duplex ultrasound and extremity segmental pressures are particularly useful source of information.

## Summary

We briefly summarised the current technologies used in CT-based FFR computations. Despite some assumptions, the efficacy of this approach has been well demonstrated, which placed it as part of routine clinical practice in many countries. Further development is expected in terms of its analysis speed, and improved accuracy by integrating with some additional parameters that could also be derived from the same set of computational results. The CT-based FFR computations also have the potential to be applied in assessing the functional severity of stenosis in other parts of the cardiovascular system as far as its technical characteristics are concerned. However, clearer clinical evidences showing the efficacy of FFR in those applications are needed, which is clearly established in the case of coronary FFR.
